# Healthcare resource utilization patterns in psoriasis patients using biologic and conventional treatments in Finland

**DOI:** 10.3389/fimmu.2024.1374829

**Published:** 2024-06-10

**Authors:** Aino Vesikansa, Juha Mehtälä, Jaakko Aaltonen, Riikka Konttinen, Kaisa Tasanen, Laura Huilaja

**Affiliations:** ^1^ MedEngine Oy, Helsinki, Finland; ^2^ AbbVie Oy, Helsinki, Finland; ^3^ Department of Dermatology and Medical Research Center, Oulu University Hospital, PEDEGO Research Unit, University of Oulu, Oulu, Finland

**Keywords:** biologic, biological treatment, healthcare resource utilization, oral immunosuppressants, Psoriasis vulgaris, real-world evidence

## Abstract

**Introduction and aim:**

Psoriasis vulgaris is associated with a significant healthcare burden, which increases over time as the disease progresses. The aim of this retrospective, population-based registry study was to characterize healthcare resource utilization (HCRU) in patients with psoriasis using biologics and oral immunosuppressants (conventionals) in Finland.

**Materials and methods:**

The study cohort included all patients with a diagnosis of psoriasis vulgaris in the secondary healthcare setting between 2012–2018, who initiated a biologic (n=1,297) or conventional (n=4,753) treatment between 2013–2017. Data on primary and secondary HCRU were collected from nationwide healthcare registries.

**Results:**

The results indicated a remarkable decrease in contacts with a dermatologist after the treatment initiation among patients starting biologic (mean annual number of contacts 5.4 per person before and 2.3 after the initiation), but not conventional (3.3 and 3.2) treatment. For conventional starters there was a high level of contacts with a dermatologist surrounding times of treatment switching, which was not observed for biologic starters.

**Conclusion:**

Overall, primary and other secondary care contacts did not decrease after the initiation or switch of treatment. The results highlight the importance of thorough consideration of the most optimal treatment alternatives, considering the overall disease burden to patients and healthcare systems.

## Introduction

Psoriasis is a chronic, immune-mediated, inflammatory skin disease, which generally affects people of working age (1). Psoriasis vulgaris (henceforth psoriasis) is the most common form of the disease, accounting for more than 80% of psoriasis cases (1). The disease is associated with an increased risk of developing comorbidities, such as psoriatic arthritis (PsA), metabolic syndrome, cardiovascular diseases, rheumatoid arthritis (RA) and inflammatory bowel disease (IBD) (2, 3).

Several studies have demonstrated that psoriasis is associated with a significant healthcare burden, which increases over time as the disease progresses (4–6). Due to its chronicity and high prevalence (1–5% of the population in Europe), psoriasis is considered one of the costliest dermatological diseases (7–9). Patients with psoriasis use more healthcare resources not only in the specialty area of dermatology, but they also experience a higher healthcare resource utilization (HCRU) and economic burden of comorbidities compared to the general population with the same comorbidities (10).

The introduction of biologics targeting the immune-mediated pathways of psoriasis has provided a significant therapeutic advancement in the treatment of moderate to severe psoriasis. Biologics inhibiting the tumor necrosis factor–α (TNF–α), interleukin (IL) -12/23, IL-17 and IL-23, as well as a small molecule inhibitor of phosphodiesterase 4 (PDE4), provide an efficacious alternative to broadly-acting oral immunosuppressants (conventionals) that have been considered the primary systemic medications for decades (1, 11, 12). However, discontinuation and switching among biologics are common in real-world clinical practice (13, 14). In the studies based on the US and Japanese databases, switching has been shown to result in higher HCRU and direct costs than remaining on the same biologic (15, 16).

In Finland, the treatment of psoriasis follows the uniform practices determined by the Current Care Guidelines (17). Biologics can be used for patients with severe psoriasis who have not responded to first-line conventionals or phototherapy. However, the order in which individual biologics should be taken is not defined. During 2012–2018, approximately 29% of psoriasis patients identified in the Finnish secondary care register (representing patients with moderate to severe disease) used conventionals and 7% used biologics (18). However, real-world data on the overall HCRU patterns associated with different treatment options is lacking.

The aim of this retrospective, population-based registry study was to characterize HCRU patterns in patients with psoriasis using biologics and conventionals in Finland. The HCRU in different care categories (primary care, dermatology, and secondary care excluding dermatology) was assessed before and after the initiation and switch of the biologics/conventionals, and by subgroups of treatment non-switchers, switchers, and discontinuers.

## Materials and methods

### Study cohort, data collection, and subgroups

**Figure 1:**
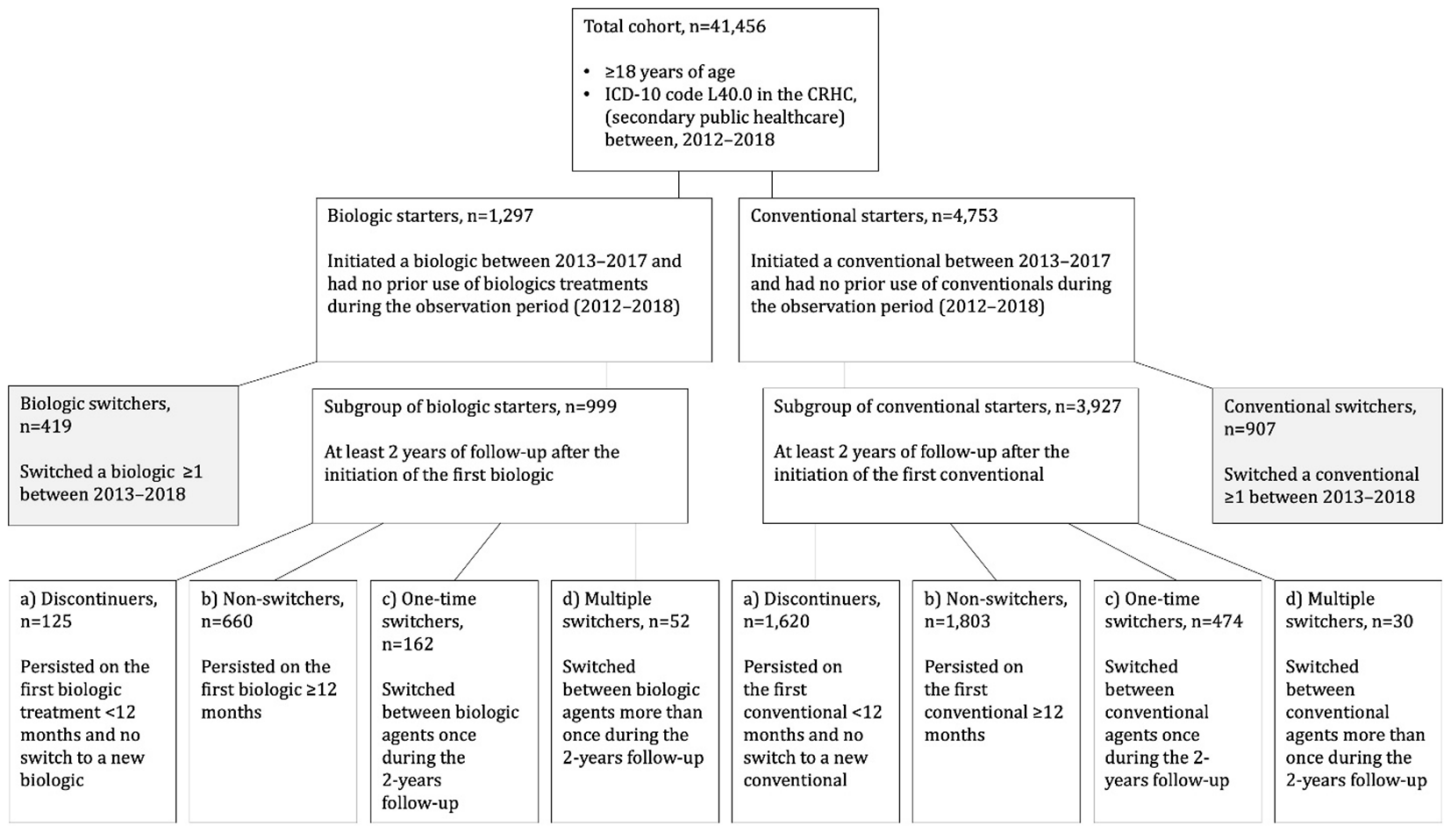
Patient flow. ICD-10, International Classification of Diseases, Tenth Revision.

For this retrospective, register-based study, all adult patients (≥18 years of age) with a diagnosis of psoriasis (International Classification of Diseases, Tenth Revision [ICD-10], diagnosis code L40.0) in the Finnish Care Register for Health Care (CRHC, secondary public healthcare) between January 1, 2012, and December 31, 2018, were identified (**Figure 1**). Individual-level data on the use of public healthcare services and diagnoses were collected from the CRHC and the Register of Primary Health Care Visits, and medication data from the Register of Reimbursed Drugs, registers with national coverage, as described in detail in (18).

For the primary analyses, the total cohort was divided into two main study groups based on purchases of reimbursable drugs from community pharmacies. The main study groups included patients who initiated A) biologic, and B) conventional during the period from January 2013 to December 2017 and had no prior use of A) biologics, and B) conventionals during the observation period (January 2012 onward; ≥12 months clean period) (**Figure 1**; **Supplementary Table S1**, and **Supplementary Methods**). The subgroup analyses included biologic and conventional starters who had at least 2 years of follow-up (**Figure 1**; **Supplementary Methods**). Additionally, for the subgroup of biologic starters and for the subgroup of conventional starters, further sub-cohorts were identified based on discontinuation, persistence or switching of biologic/conventional therapy. Subgroups were defined as patients who, a) were on treatment for <12 months from the initiation (discontinuers), b) persisted on a single treatment for ≥12 months (non-switchers), and c) switched a biologic or conventional once during the 2-year period after the initiation of the first treatment (one-time switchers, total treatment duration ≥12 months), and d) switched a biologic or conventional more than once during the 2-year period after the initiation of the first treatment (multiple switchers, total treatment duration ≥12 months). The subgroup follow-up started one year before and ended two years after the initiation of the first biologic/conventional (**Figure 1**).

### Outcome measures

The two outcome measures of the study were 1) the mean annual number of healthcare contacts (including all contact types, e.g. visits and phone calls) per person before and after the initiation/switch of the treatment in biologic and conventional starters, and 2) the mean and cumulative number of healthcare contacts per person one year before to two years after the initiation of the first treatment in the subgroups of biologic and conventional starters. The healthcare contacts were reported as number of days the patient had any contact with a healthcare provider, divided in the following categories: primary care, dermatology specialty in secondary care, and other secondary care excluding dermatology. Treatment switch was defined as the purchase of a new biologic/conventional drug (analyzed in the Anatomical Therapeutic Chemical Classification System (ATC) classes at the level of 7 digits) per time window.

### Statistical analyses

Demographic characteristics and comorbidities were analyzed using descriptive statistics. Categorical variables were presented as the number of observations and proportions. Continuous variables were reported as mean with standard deviation (SD) and median with first (Q1) and third (Q3) quartiles.

The outcomes were illustrated by figures describing a moving average as a function of time from the index date. In addition, the outcomes were described by the number of events, accumulated person-years, and rate of events (number of events per person-years) before and after the index date. The rate of events was compared using the Poisson model.

The statistical analyses were conducted using R (version 4.1.3., http://www.r-project.org).

## Results

### Characteristics of biologic and conventional starters

During the observation period, the initial treatment was a biologic in 3.1% (n=1,297) of patients, and conventional in 11.5% (n=4,753) of patients (**Figure 1**). The baseline demographic characteristics and comorbidities for the main study groups are presented in **Table 1**. Biologic starters were slightly more often male (63.7%) than conventional starters (58.2%) and the mean age was younger for biologic starters (51.8 years; SD, 13.8 vs. 56.5 years; SD, 15.5). Immune-mediated inflammatory comorbidities, such as psoriatic arthropathies, rheumatoid arthritis, and inflammatory bowel diseases were more common in biologic compared with conventional starters, whereas other dermatological diseases were more common in conventional starters.

**Table 1:** Characterization of biologic (n=1,297) and conventional (n=4,753) starters.

	Biologic starters (n=1,297)	Conventional starters (n=4,753)
Sex, n (%)		
*Female*	471 (36.3)	1,986 (41.8)
*Male*	826 (63.7)	2,767 (58.2)
Age – continuous		
*Mean (SD)*	51.83 (13.84)	56.48 (15.51)
*Median*	52.58	57.88
*Q1, Q3*	41.80, 62.11	45.48, 68.16
Selected comorbidities, n (%) (based on ICD-10 codes from 2012–2018)		
*Essential (primary) hypertension*	226 (17.4)	960 (20.2)
*Arthropathy*	285 (22.0)	1,118 (23.5)
*Distal interphalangeal psoriatic arthropathy*	39 (3.0)	48 (1.0)
*Arthropathic psoriasis*	590 (45.5)	1,015 (21.4)
*Dorsopathies*	297 (22.9)	959 (20.2)
*Acute upper respiratory infections*	215 (16.6)	749 (15.8)
Other dermatological diseases*	151 (11.6)	910 (19.1)
*Type 2 diabetes mellitus*	127 (9.8)	521 (11.0)
*Osteoarthritis*	132 (10.2)	539 (11.3)
*Severe ischemic arrhythmias*	61 (4.7)	323 (6.8)
*Hypercholesterolemia*	83 (6.4)	18 (0.4)
*Influenza and pneumonia*	69 (5.3)	235 (4.9)
*Other lower respiratory infections*	89 (6.9)	375 (7.9)
*Cancer*	33 (2.5)	293 (6.2)
*Asthma*	80 (6.2)	322 (6.8)
*Major depressive disorder*	109 (8.4)	351 (7.4)
*Any mental disorder*	222 (17.1)	844 (17.8)
*Heart failure*	24 (1.9)	91 (1.9)
*Hemorrhagic or embolic stroke*	13 (1.0)	110 (2.3)
*Chronic obstructive pulmonary disease*	29 (2.2)	144 (3.0)
*Gout*	23 (1.8)	119 (2.5)
*Kidney diseases*	13 (1.0)	44 (0.9)
*Crohn’s disease*	35 (2.7)	56 (1.2)
*Ulcerative colitis*	27 (2.1)	66 (1.4)
*Crohn’s disease or ulcerative colitis*	54 (4.2)	111 (2.3)
*Rheumatoid arthritis*	90 (6.9)	173 (3.6)

ICD-10, International Classification of Diseases, Tenth Revision; Q1, first quartile; Q3, third quartile; SD, standard deviation. *ICD-10 codes: L20–9, L30–9.

### HCRU before and after the initiation or switch of a biologic

**Figure 2:**
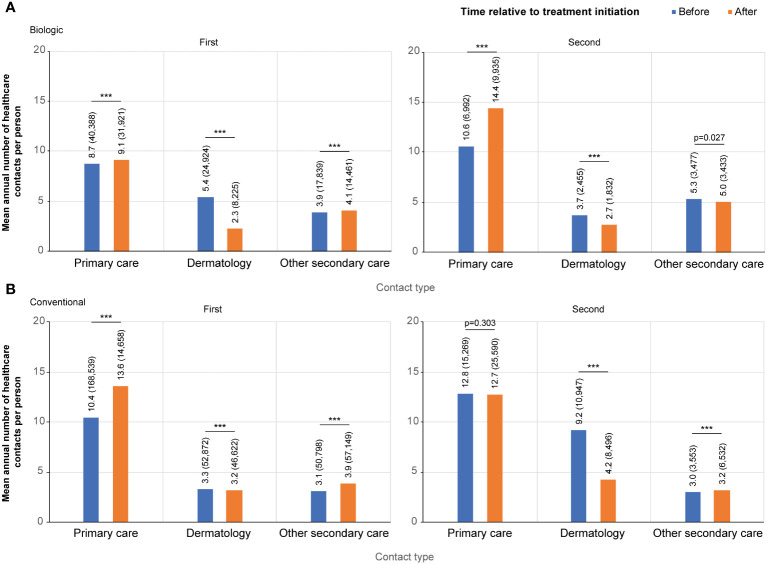
Contact rates per category before and after the initiation of the first and the second **(A)** biologic, and **(B)** conventional. Contact categories included: all primary care, dermatology, and other secondary care excluding dermatology. For the first biologic/conventional, the follow-up starts at January 1, 2012, and ends either when a patient switches to a second biologic or December 31, 2018, whichever occurred first (the index date is the date of the first drug initiation). For the second biologic/conventional, the follow-up starts from the initiation of a first biologic/conventional and ends when a patient switches to a third biologic, or on December 31, 2018, whichever occurred first (the index date is the date of the treatment switch). *** p<0.001.

The mean annual number of contacts with a dermatologist decreased from 5.4 to 2.3 per person after the initiation of the first biologic (n=1,297) (**Figure 2A**). The mean annual number of both primary care (8.7 and 9.1 per person before and after the initiation, respectively) and other secondary care (3.9 and 4.1) contacts was higher after the initiation of the first biologic (**Figure 2A**). In all categories, the number of contacts peaked just before the initiation of biologic treatment (**Supplementary Figure S1A**).

A total of 419 patients (32.3%) switched biologic ≥1 time during the observation period. The peak in the contacts with a dermatologist was lower at the time of treatment switch than at the initiation of the first biologic (**Supplementary Figure S2A**, **Supplementary Figure S1A**), as was the difference between the mean number of contacts before (3.7 per person per year) and after (2.7) the treatment switch compared to the initiation of the first biologic (**Figure 2A**).

### HCRU before and after the initiation or switch of a conventional

For the conventional starters (n=4,753), the mean annual number of contacts with a dermatologist was 3.3 per person before and 3.2 after the initiation of the first conventional, with a high, symmetric peak in contacts at the time of treatment initiation (**Figure 2B**; **Supplementary Figure S1B**). The mean annual number of both other secondary (3.1 and 3.9 per person before and after the index, respectively) and primary care contacts (10.4 and 13.6) increased after the initiation of the first conventional (**Figure 2B**).

A total of 907 (19%) patients switched ≥1 time among conventionals during the observation period. For these patients, the mean annual number of contacts with a dermatologist before the treatment switch was almost three times higher (9.2 vs 3.3 per person) than before the initiation of the first conventional (**Figure 2B**; **Supplementary Figure S2B**). After the conventional switch, the annual number of contacts with a dermatologist decreased to an annual mean of 4.2 per person.

### HCRU in the subgroups of biologic starters

HCRU from one year before to two years after the initiation of the first biologic was analyzed in the subgroups of patients who had ≥2 years of follow-up (n=999) (**Figure 1**). A total of 66.1% (n=660) of patients persisted on the first biologic and 12.5% (n=125) discontinued the treatment during the 12 months following the treatment initiation. During the 2-year period after the initiation of the first biologic, 16.2% (n=162) patients switched a biologic once and 5.2% (n=52) more than once.

**Figure 3:**
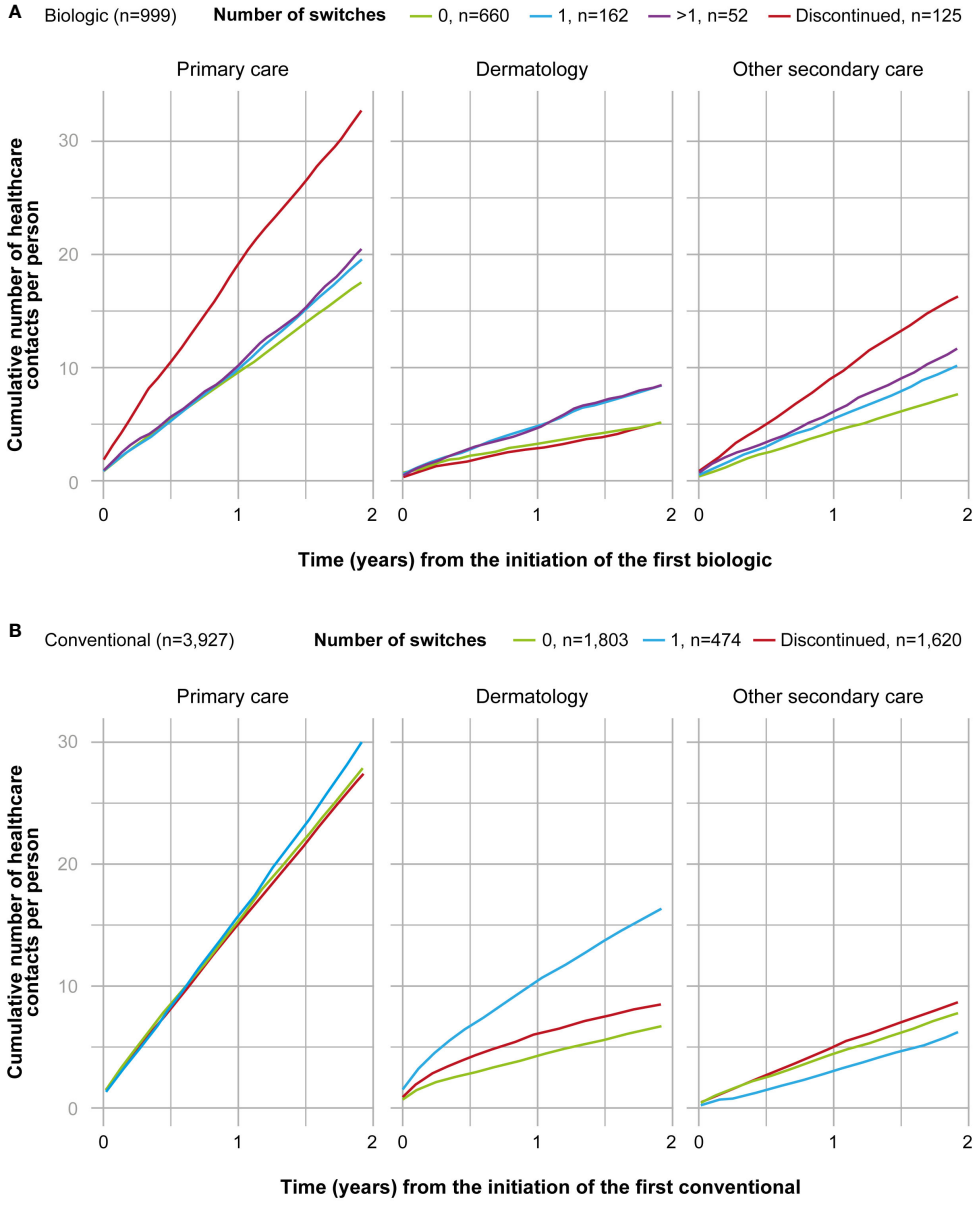
Cumulative number of healthcare contacts from the 2-year period after the initiation of the first **(A)** biologic (n=999), and **(B)** conventional (n=3,927) in different subgroups and by category (primary care, dermatology, secondary care excluding dermatology). Subgroups of biologic starters included patients who persisted on the first biologic ≥12 months (0, n=660); switched the biologic once during the 2-years period (1, n=162); switched the biologic more than once during 2-years period (>1, n=52); and patients who persisted on the first biologic <12 months (discontinued, n=125). Subgroups of conventional starters included patients who persisted on the first conventional <12 months (discontinued, n=1,620); persisted on the first conventional ≥12 months (0, n=1,803); and switched the conventional once during the 2-year period (1, n=474). Patients who switched conventional more than once during the 2-year period were excluded from the figure due to the small number (>1, n=30).

**Figure 4:**
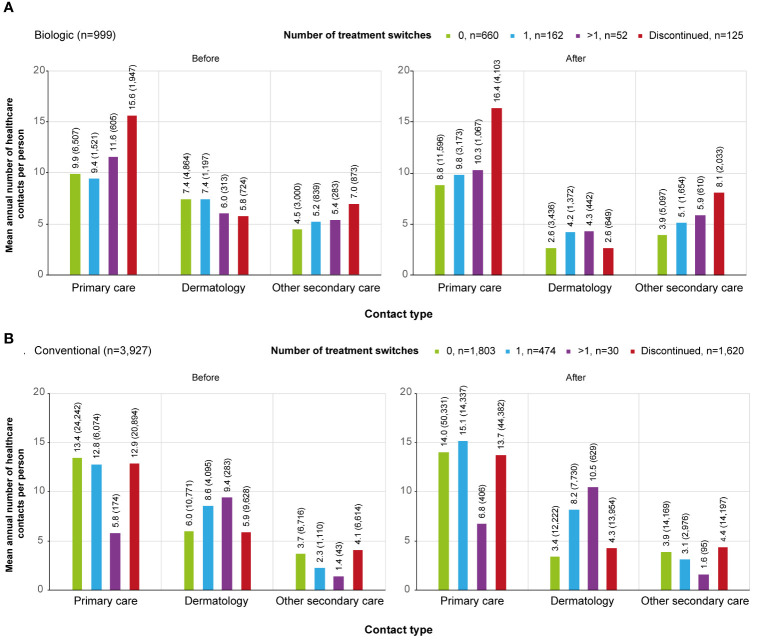
Contact rates by category (primary care, dermatology, secondary care excluding dermatology) before and after the initiation of the first **(A)** biologic / **(B)** conventional in different subgroups.. Subgroups were defined as follows, patients who: persisted on the first treatment ≥12 months (0, biologic n=660, conventional n=1,803); switched a biologic or conventional once during the 2-year period after the initiation of the first treatment (1, biologic n=162, conventional n=474); switched a biologic or conventional more than once during the 2-year period after the initiation of the first treatment (>1, biologic n=52, conventional n=30); continued the first treatment <12 months from the initiation and did not start a new medication within the medication group (discontinued, biologic n=125, conventional n=1,620).

The cumulative number of healthcare contacts during the 2-year period was significantly lower for non-switchers compared to all other subgroups in all care categories excluding contacts with a dermatologist for patients who discontinued the treatment (p<0.001) (**Figure 3A**). In non-switchers, the mean number of annual contacts with a dermatologist in the year before the initiation of the first biologic was 7.4 per person, compared to 2.6 during the 2-year period after the initiation (n=660) (**Figure 4A**). In non-switchers, the mean annual number of other secondary care and primary care contacts also decreased after the treatment initiation.

For the discontinuers, the mean number of primary and other secondary care contacts was significantly higher than for any other subgroup during the 2-year period (p<0.001). The difference was observed before the initiation of the first biologic (**Figure 4A**). In discontinuers, but not in any other subgroup, Crohn´s disease (recorded in 5% of all secondary care visits) and rheumatoid arthritis (4%) appeared as one of the most common reasons for visits (**Supplementary Table S2**).

### HCRU in the subgroups of conventional starters

Of the conventional starters who had ≥2 years of follow-up (n=3,927), 45.9% (n=1,803) persisted on the first conventional treatment and 41.3% (n=1,620) discontinued the treatment during the first year after the initiation (**Figure 1**). During the 2-year period after initiation of the first conventional, 12.1% (n=474) switched the treatment once, and only 30 (1%) switched more than once.

The cumulative number of contacts during the 2-year period after the initiation of the first conventional was significantly (p<0.001) different between all subgroups and care categories, excluding the discontinuers (n=1,620) and non-switchers (n=1,803) in primary care contacts (**Figure 3B**). In the non-switchers, the mean annual number of contacts with a dermatologist decreased after the initiation of the first conventional (6.0 and 3.4. per person before and after the initiation, respectively; n=1,803) whereas for patients switching once, it remained at a similar level (8.6 and 8.2; n=474) (**Figure 4B**).

## Discussion

This study characterized the HCRU patterns in the population-based cohort of psoriasis patients using systemic treatments in Finland. The results showed a high overall HCRU burden consisting not only of contacts with a dermatologist, but also primary and other secondary care HCRU. A decrease in contacts with a dermatologist was observed after treatment initiation among patients who initiated a biologic, but not a conventional. Treatment switching correlated with steep peak in contacts with a dermatologist for conventional users, but not for those using a biologic treatment. Overall, primary and other secondary care contacts besides dermatology contacts did not decrease after the initiation or switch of either biologic or conventional treatments.

Previous studies have shown that the initiation of biologics is associated with a significant decrease in HCRU and associated costs in patients with moderate to severe psoriasis (19–21). Some of the first studies have suggested that the decrease in HCRU after the initiation of the biologics can even offset the higher prescription costs associated with biologics (19). However, most of the previous studies are based either on the US claims databases or small cohorts in Europe – nationally representative, population-based analyses are rare. In line with the previous findings, this study indicated a significant decrease in the mean annual number of contacts with a dermatologist in the period after (2.3 per person) compared to before the initiation of biologics (5.4). The observed decrease, specifically in the contacts with a dermatologist, likely reflects improved psoriasis control resulting in fewer hospitalizations and emergency room visits, as previously shown, but also suggests a need for less frequent control visits (21, 22).

Interestingly, only a very slight decrease in the number of contacts with a dermatologist (3.3 before and 3.2 after the initiation, respectively) was observed in the conventional starters after the initiation of the first conventional. The mean number of contacts with a dermatologist after the treatment initiation was higher in the conventional starters compared to biologic starters, even though conventional starters likely suffer from less severe disease. According to the Finnish Current Care Guidelines for Psoriasis and the reimbursement criteria by the Social Insurance Institution of Finland, biologics can be used only for patients with severe psoriasis who have not responded or are intolerant to first-line systemic treatments or phototherapy (17). This is notable from a healthcare perspective in general, as a vast majority of systemic users are on conventional treatment, causing the most significant burden on healthcare providers (18).

Limited treatment persistence has been reported with biologics, with one-year persistence rates ranging from 30% to 70% in different studies (13, 14, 16, 23, 24). Although switching of biologic agents due to inefficacy or adverse effects can improve disease control, switching and discontinuing the treatment have been reported to be associated with a significant healthcare burden compared to continuing treatment on the same biologic (15). In this study, 21% of biologic and 13% conventional starters switched between agents, and 13% of biologic and 41% of conventional starters discontinued the treatment during the first two years after the initiation of their first biologic/conventional. Switching to the second biologic caused a smaller peak in the contacts with a dermatologist compared to the initiation of the first biologic, suggesting that the switch as such is not associated with a considerable HCRU burden.

Instead, there was a significant peak in contacts with a dermatologist before switching to the second conventional, and the 2-year cumulative numbers of contacts with a dermatologist were approximately 2.5 times higher for conventional switchers vs. non-switchers. These findings suggest that for the subgroup of conventional switchers, switching to a biologic instead of another conventional could be a potential option to decrease the burden to both patients and healthcare system.

Although the switch to the second biologic did not correspond with a peak in the average annual contacts with a dermatologist, the cumulative healthcare burden was significantly lower for biologic non-switchers than for switchers in all care categories studied, in line with previous findings (15, 16). The non-switchers were the only subgroup in which the average number of contacts decreased in all care categories including primary and other secondary care, suggesting that improved psoriasis control may have favorable effects on overall HCRU even with a relatively short timeframe.

Based on the HCRU pattern, biologic discontinuers seemed to differ from those persisting or switching treatment. The discontinuers had a higher number of primary and other secondary care contacts before the initiation of the biologic, suggesting a higher burden of comorbidities in these patients. In fact, previous studies have indicated that patients with a high comorbidity index and concomitant medications had an increased risk of biologic discontinuation (13, 25, 26). Another possible explanation is that among the discontinuers, biologics are more often used for another indication than psoriasis. This idea is supported by the finding that the number of contacts with a dermatologist was relatively low in the discontinuers, while other inflammatory diseases such as Crohn´s disease and rheumatoid arthritis appeared as one of the most common reasons for visits, specifically in biologic discontinuers.

The major strengths of this population-based study include utilization of nationwide healthcare registers providing a representative picture of the patient population at a national level. The Finnish healthcare system allows all citizens equal access to tax-funded, high-quality public healthcare with an annual maximum limit on out-of-pocket costs, minimizing the selection bias in due to accessibility reasons. This is especially important regarding biologic treatments, for which the costs are globally one of the major factors limiting accessibility to patients.

Limitations of the study include a lack of detailed clinical information data and indication of biologics, as well as reasons for treatment discontinuation and switches. Another limitation is the fact that national registries used in this study are not quality registries per se, and therefore, lack disease-specific data such as Psoriasis Area and Severity Index. Incorporation of disease-specific structural parameters into registries would allow even more comprehensive analyses. In addition, the use of private and occupational healthcare is not recorded in the national health registers. Private care accounted for approximately 22% of all healthcare provided in Finland in 2020, thus the actual use of healthcare services is higher than what was reported here (27). This applies especially to primary care, whereas most of the secondary care is organized by public healthcare service providers in Finland. Analyses on systemic treatments were based on purchases of nationally reimbursed prescription medicines, and thus do not include drugs administered in hospitals, such as intravenous infliximab. Although the study design includes only a minimal bias in patient selection, and the results are thus expected to reflect moderate-to-severe psoriasis patients in general, it should be noted that healthcare systems and treatment practices may vary considerably between countries. Moreover, patients who start biologics have used conventional treatments before, since in Finland biologics are reimbursed only after use of conventionals. Therefore, from the analysis point of view, it is possible that patients that are conventional discontinuers are also contemplated in the biologic starters group, which brings some limitations for the conventional discontinuers analysis.

With the increasing number of treatment options available for psoriasis, identification of patients’ individual needs and preferences and understanding of disease burden comprehensively become more important than ever. This study provides a nationwide real-world view on the HCRU in psoriasis patients using systemic treatments. The results strengthen previous evidence on the benefits of biologics in decreasing the HCRU and associated costs in psoriasis patients, however, the benefits reach beyond that. Less frequent healthcare visits have a positive impact on patients in terms of reduced days off from work, improved work productivity, and overall activity in psoriasis patients, decreasing the burden and indirect costs of the disease (13, 27–31). The results highlight the importance of thorough consideration of the most optimal treatment alternatives, considering the overall disease burden to patients and healthcare systems. This includes the repertoire of new biologics, and the possibility to switch, especially in patients not responding to the first conventional treatment.

## Data availability statement

The datasets presented in this article are not readily available because according to Finnish legislation, access to individual-level data is restricted only to individuals named in the study permit. The study protocol is available upon request from the corresponding author.

## Ethics statement

Ethical approval was not required for the study involving humans in accordance with the local legislation and institutional requirements. Written informed consent to participate in this study was not required from the participants or the participants’ legal guardians/next of kin in accordance with the national legislation and the institutional requirements. Written informed consent was not obtained from the individual(s) for the publication of any potentially identifiable images or data included in this article because the datasets presented in this article are not readily available (according to Finnish legislation, access to individual-level data is restricted only to individuals named in the study permit).

## Author contributions

AV: Conceptualization, Data curation, Investigation, Methodology, Project administration, Validation, Visualization, Writing – original draft, Writing – review & editing. JM: Data curation, Investigation, Methodology, Validation, Visualization, Writing – review & editing, Conceptualization. JA: Conceptualization, Investigation, Methodology, Project administration, Writing – review & editing. RK: Conceptualization, Investigation, Methodology, Writing – review & editing. KT: Conceptualization, Investigation, Methodology, Writing – review & editing. LH: Conceptualization, Investigation, Methodology, Validation, Writing – review & editing.

## References

[1] Armstrong AW Read C. Pathophysiology, clinical presentation, and treatment of psoriasis: A review. JAMA. (2020) 323:1945–60. doi: 10.1001/jama.2020.4006 32427307

[2] Augustin M Reich K Glaeske G Schaefer I Radtke M. Co-morbidity and age-related prevalence of psoriasis: Analysis of health insurance data in Germany. Acta Derm Venereol. (2010) 90:147–51. doi: 10.2340/00015555-0770 20169297

[3] Yeung H Takeshita J Mehta NN Kimmel SE Ogdie A Margolis DJ. Psoriasis severity and the prevalence of major medical comorbidity: a population-based study. JAMA Dermatol. (2013) 149:1173–9. doi: 10.1001/jamadermatol.2013.5015 PMC3800487 23925466

[4] Armstrong AW Foster SA Comer BS Lin CY Malatestinic W Burge R. Real-world health outcomes in adults with moderate-to-severe psoriasis in the United States: a population study using electronic health records to examine patient-perceived treatment effectiveness, medication use, and healthcare resource utilization. BMC Dermatol. (2018) 18:4. doi: 10.1186/s12895-018-0072-2 29954363 PMC6025830

[5] Al Sawah S Foster SA Goldblum OM Malatestinic WN Zhu B Shi N. Healthcare costs in psoriasis and psoriasis sub-groups over time following psoriasis diagnosis. J Med Econ. (2017) 20:982–90. doi: 10.1080/13696998.2017.1345749 28635342

[6] Feldman SR Zhao Y Shi L Tran MH. Economic and comorbidity burden among patients with moderate-to-severe psoriasis. J Manag Care Spec Pharm. (2015) 21:874–88. doi: 10.18553/jmcp.2015.21.10.874 PMC10397856 26402388

[7] Levy AR Davie AM Brazier NC Jivraj F Albrecht LE Gratton D. Economic burden of moderate to severe plaque psoriasis in Canada: Economic burden of moderate to severe psoriasis in Canada. Int J Dermatol. (2012) 51:1432–40. doi: 10.1111/j.1365-4632.2011.05359.x 23171010

[8] Parisi R Iskandar IYK Kontopantelis E Augustin M Griffiths CEM Ashcroft DM. National, regional, and worldwide epidemiology of psoriasis: systematic analysis and modelling study. BMJ. (2020) 369:m1590. doi: 10.1136/bmj.m1590 32467098 PMC7254147

[9] Brezinski EA Dhillon JS Armstrong AW. Economic burden of psoriasis in the United States: A systematic review. JAMA Dermatol. (2015) 151:651–8. doi: 10.1001/jamadermatol.2014.3593 25565304

[10] Wu JJ Suryavanshi M Davidson D Patel V Jain A Seigel L. Economic burden of comorbidities in patients with psoriasis in the USA. Dermatol Ther. (2023) 13:207–19. doi: 10.1007/s13555-022-00832-9 PMC9823180 36402940

[11] Sbidian E Chaimani A Garcia-Doval I Do G Hua C Mazaud C. Systemic pharmacological treatments for chronic plaque psoriasis: a network meta-analysis. Cochrane Database Syst Rev. (2017) 12:CD011535. doi: 10.1002/14651858.CD011535.pub2 29271481 PMC6486272

[12] Rønholt K Iversen L. Old and new biological therapies for psoriasis. Int J Mol Sci. (2017) 18:2297. doi: 10.3390/ijms18112297 29104241 PMC5713267

[13] Mahlich J Alba A Hadad LE Leisten MK Peitsch WK. Drug survival of biological therapies for psoriasis treatment in Germany and associated costs: A retrospective claims database analysis. Adv Ther. (2019) 36:1684–99. doi: 10.1007/s12325-019-00969-8 31102203

[14] Doshi JA Takeshita J Pinto L Li P Yu X Rao P. Biologic therapy adherence, discontinuation, switching, and restarting among patients with psoriasis in the US Medicare population. J Am Acad Dermatol. (2016) 74:1057–1065.e4. doi: 10.1016/j.jaad.2016.01.048 26946986 PMC4945117

[15] Feldman SR Tian H Wang X Germino R. Health care utilization and cost associated with biologic treatment patterns among patients with moderate to severe psoriasis: analyses from a large U. S. Claims Database. J Manag Care Spec Pharm. (2019) 25:479–88. doi: 10.18553/jmcp.2018.18308 PMC10398133 30556761

[16] Tada Y Kim H Spanopoulos D Habiro K Tsuritani K Yamada Y. Treatment patterns, healthcare resource utilization, and costs in patients with moderate-to-severe psoriasis treated with systemic therapy in Japan: A retrospective claims database study. J Dermatol. (2022) 49:1106–17. doi: 10.1111/1346-8138.16543 PMC9804179 35946343

[17] Working group set up by the Finnish Medical Society Duodecim, the Finnish Dermatological Society and the FS for Rheumatology. Psoriasis and Psoriatic Arthritis, in: Current care guidelines. Available online at: https://www.kaypahoito.fi/hoi50062duo-Informpractice (Accessed 2023 May 31).

[18] Vesikansa A Mehtälä J Pesu M Aaltonen J Konttinen R Tasanen K. Comorbidities and medication use in finnish patients with psoriasis: A population-based registry study. . Acta Derm Venereol. (2023) 103:adv00886. doi: 10.2340/actadv.v103.3491 36892511 PMC10012470

[19] Bhosle MJ Feldman SR Camacho FT Timothy Whitmire J Nahata MC Balkrishnan R. Medication adherence and health care costs associated with biologics in Medicaid-enrolled patients with psoriasis. J Dermatol Treat. (2006) 17:294–301. doi: 10.1080/09546630600954594 17092860

[20] Fonia A Jackson K LeReun C Grant DM Barker JNWN Smith CH. A retrospective cohort study of the impact of biologic therapy initiation on medical resource use and costs in patients with moderate to severe psoriasis: Biologic therapy: medical resource use and costs. Br J Dermatol. (2010) 163:807–16. doi: 10.1111/j.1365-2133.2010.09944.x 20662837

[21] Degli Esposti L Perrone V Sangiorgi D Buda S Andretta M Rossini M. Analysis of drug utilization and health care resource consumption in patients with psoriasis and psoriatic arthritis before and after treatment with biological therapies. Biol Targets Ther. (2018) 12:151–8. doi: 10.2147/BTT PMC6237141 30518996

[22] Rodriguez-Jato Q Pereira A Batalla A Abalde M Salgado-Boquete L Martinez-Reglero C. Hospitalization in patients with psoriasis: impact of biological therapies on temporal evolution. J Drugs Dermatol. (2021) 20:208–14. doi: 10.36849/JDD 33538560

[23] Navarini AA French LE. Survival of second-line biologics in psoriasis: the british BADBIR registry data informs daily practice. J Invest Dermatol. (2018) 138:726–8. doi: 10.1016/j.jid.2018.02.008 29579453

[24] Mourad AI Gniadecki R. Biologic drug survival in psoriasis: A systematic review & Comparative meta-analysis. Front Med. (2021) 18:7: 625755. doi: 10.3389/fmed.2020.625755 PMC8012481 33816514

[25] Iskandar IYK Warren RB Lunt M Mason KJ Evans I McElhone K. Differential drug survival of second-line biologic therapies in patients with psoriasis: observational cohort study from the british association of dermatologists biologic interventions register (BADBIR). J Invest Dermatol. (2018) 138:775–84. doi: 10.1016/j.jid.2017.09.044 PMC5869053 29080680

[26] Özkur E Kıvanç Altunay İ Oğuz Topal İ Aytekin S Topaloğlu Demir F Özkök Akbulut T. Switching biologics in the treatment of psoriasis: A multicenter experience. Dermatology. (2021) 237:22–30. doi: 10.1159/000504839 31865339

[27] Terveyden ja hyvinvoinnin laitos, Terveydenhuollon menot ja rahoitus 2020 (2021). Available online at: https://thl.fi/tilastot-ja-data/tilastot-aiheittain/sosiaali-ja-terveydenhuollon-resurssit/terveydenhuollon-menot-ja-rahoitus (Accessed 20.12.2023).

[28] Norlin J Carlsson K Persson U Schmitt-Egenolf M. Resource use in patients with psoriasis after the introduction of biologics in Sweden. Acta Derm Venereol. (2015) 95:156–61. doi: 10.2340/00015555-1895 24819980

[29] Kimball AB Yu AP Signorovitch J Xie J Tsaneva M Gupta SR. The effects of adalimumab treatment and psoriasis severity on self-reported work productivity and activity impairment for patients with moderate to severe psoriasis. J Am Acad Dermatol. (2012) 66:e67–76. doi: 10.1016/j.jaad.2010.10.020 21616560

[30] Reich K Schenkel B Zhao N Szapary P Augustin M Bourcier M. Ustekinumab decreases work limitations, improves work productivity, and reduces workdays missed in patients with moderate-to-severe psoriasis: Results from PHOENIX 2. J Dermatol Treat. (2011) 22:337–47. doi: 10.3109/09546634.2010.499931 21034290

[31] Vender R Lynde C Ho V Chau D Poulin-Costello M. Work productivity and healthcare resource utilization outcomes for patients on etanercept for moderate-to-severe plaque psoriasis: results from a 1-year, multicentre, open-label, single-arm study in a clinical setting. Appl Health Econ Health Policy. (2012) 10:343–53. doi: 10.1007/BF03261868 22877226

