# Supplementary material for: Healthcare resource utilization patterns in psoriasis patients using biologic and conventional treatments in Finland
Source: Front Immunol. 2024 Jun 10;15:1374829. doi: 10.3389/fimmu.2024.1374829 (PMC11194666; doi:10.3389/fimmu.2024.1374829)
Supplement: Supplementary file 1 [file DataSheet_1.pdf]

frontiers

Supplementary Material

Healthcare resource utilization patterns in psoriasis patients using biologic and conventional treatments in Finland

Aino Vesikansa[1], Juha Mehtälä[1], Jaakko Aaltonen[2], Riikka Konttinen[2], Kaisa Tasanen[3], Laura Huilaja[3]

[1]MedEngine Oy, Eteläranta 14, 00130 Helsinki, Finland

[2]AbbVie Oy, Pasilan Asema Aukio 1, 00520 Helsinki, Finland

[3]Department of Dermatology and Medical Research Center, Oulu University Hospital; PEDEGO Research Unit, University of Oulu, 90014 Oulu, Finland

*** Correspondence:**
Aino Vesikansa
avesikans@gmail.com

SUPPLEMENTAL METHODS

Main study groups and subgroups

Biologic starters included patients who initiated treatment with a biologic during the period from January 2013 to December 2017 and had no prior use of biologics during the observation period (January 2012 onward; ≥12 months clean period without the use of biologics) (**Figure 1**). During the observation period, biologics that were reimbursable in Finland were adalimumab, brodalumab, certolizumab pegol, etanercept, ixekizumab, secukinumab, and ustekinumab (**Table SI**). Conventional starters included patients who initiated a conventional (acitretin, cyclosporine, and methotrexate) treatment during the period from January 2013 to December 2017 and had no prior use of conventionals during the observation period (January 2012 onward; ≥12 months clean period without the use of conventionals).

SUPPLEMENTAL FIGURES AND FIGURE LEGENDS

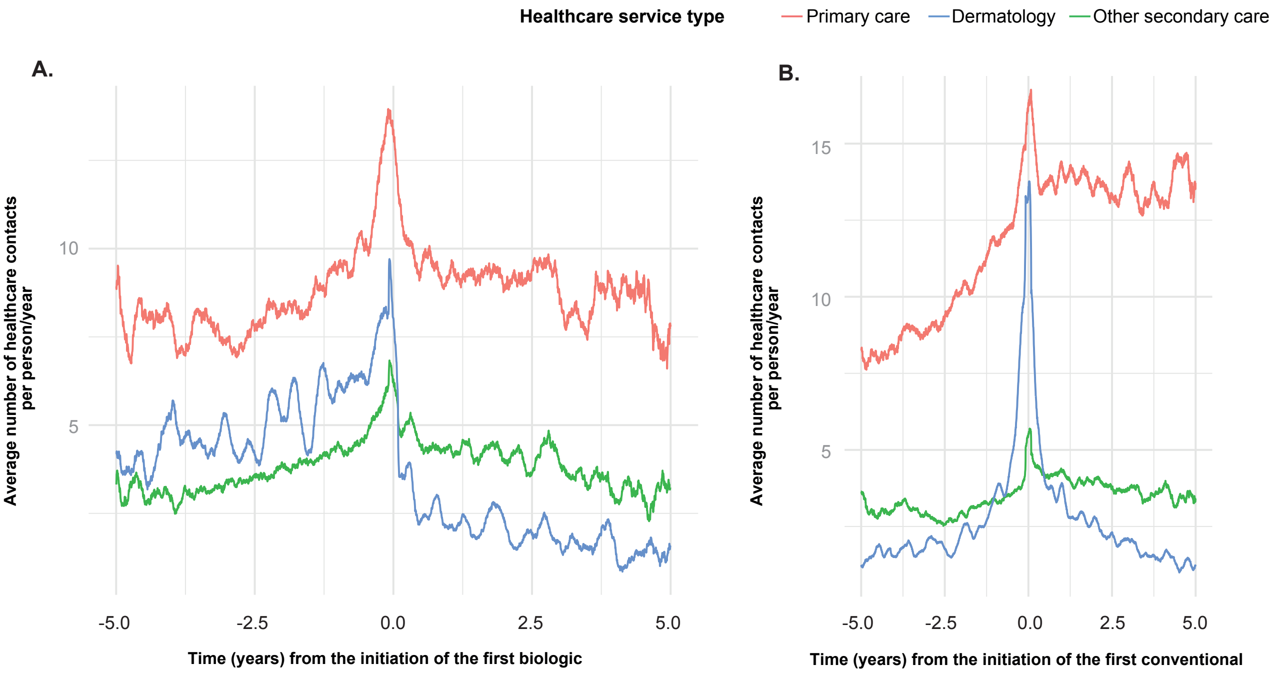

Supplementary Figure S1. All healthcare contacts (primary care, dermatology, secondary care excluding dermatology) per person per year (2 months moving average) before and after the initiation of the first A) biologic (n=1,297), and B) conventional (n=4,753). The follow-up starts at January 1, 2012, and ends when a patient switches to a second biologic/conventional, or on December 31, 2018, whichever occurred first.

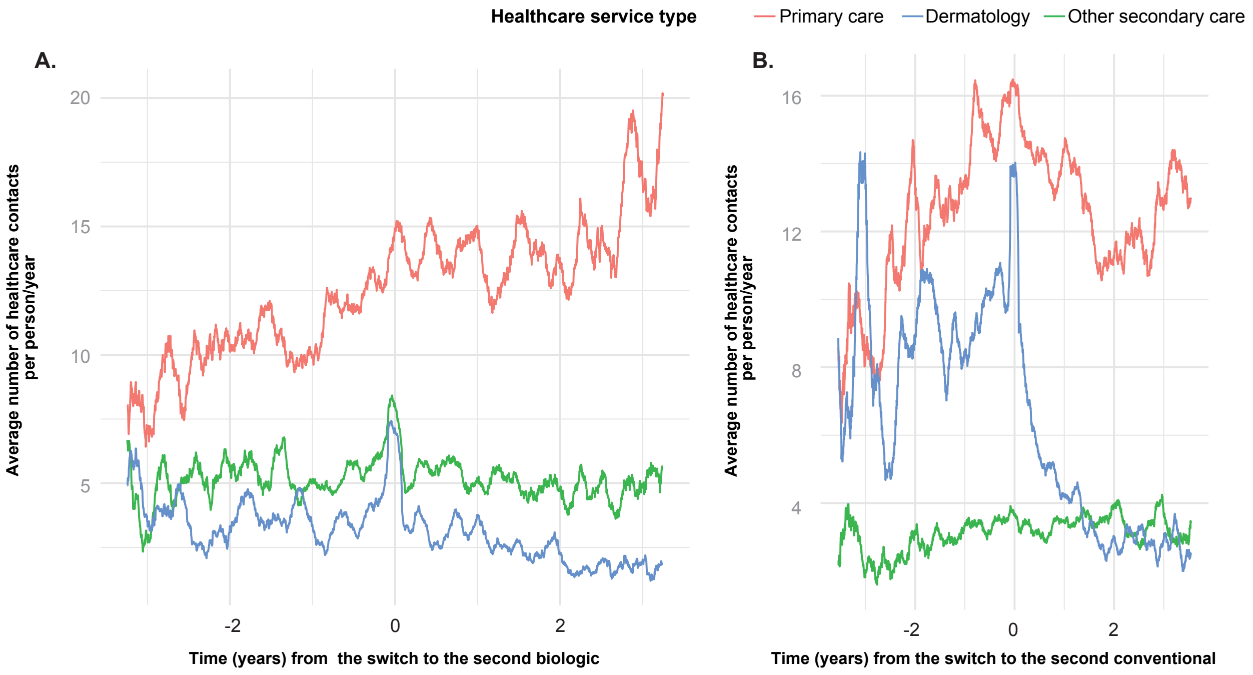

Supplementary Figure S2. All healthcare contacts (primary care, dermatology, secondary care excluding dermatology) per person per year (2 months moving average) before and after the switching to the second A) biological (n=419), and B) conventional (n=907). The follow-up starts from the initiation of a first biologic and ends when a patient switches to a third biologic/conventional, or on December 31, 2018, whichever occurred first.

SUPPLEMENTAL TABLES

Supplementary Table S1. Definitions of study treatments for psoriasis based on ATC classification codes.

Treatment	ATC code
**Conventional systemic**	
Cyclosporine	L04AD01
Methotrexate	L04AX03
Acitretin	D05BB02
**Biologic**[a]	
Adalimumab	L04AB04
Brodalumab	L04AC12
Certolizumab pegol	L04AB05
Etanercept	L04AB01
Ixekizumab	L04AC13
Secukinumab	L04AC10
Ustekinumab	L04AC05

ATC, anatomical therapeutic chemical.
[a]Biologics include treatments that were reimbursable for psoriasis vulgaris or psoriasis arthritis in Finland during the observation period. Medications do not include hospital-administered drugs.

Supplementary Table S2. The five most common reasons for secondary care visits in the A) biologic (n=999), and B) conventional (n=3,297) starters. The table shows the percentage of visits with a code during the 2-year period after the first biologic/conventional, including both primary and secondary diagnoses but excluding psoriasis diagnoses (ICD-10; L40.0) and a group of Z-codes (factors influencing health status and contact with health services). Subgroups were defined as patients who: persisted on the first treatment ≥12 months (0, biologic n=660, conventional n=1,803); switched a biologic or conventional once during the 2-year period after the initiation of the first treatment (1, biologic n=162, conventional n=474); switched a biologic or conventional more than once during the 2-year period after the initiation of the first treatment (>1, biologic n=52, conventional n=30); continued the first treatment <12 months from the initiation; and did not start a new medication within the medication group (discontinued, biologic n=125, conventional n=1,620).

Subgroup	1st	2nd	3rd	4th	5th
Biologic starters (n=999)					
0 (n=660)	M07 Psoriatic and enteropathic arthropathies (13%)	F32 Depressive episode (4%)	I10 Essential (primary) hypertension (3%)	N03 Chronic nephritic syndrome (3%)	N18 Chronic kidney disease (3%)
1 (n=162)	M07 Psoriatic and enteropathic arthropathies (25%)	I10 Essential (primary) hypertension (3%)	F33 Major depressive disorder, recurrent (3%)	E11 Type 2 diabetes mellitus (3%)	G47 Sleep disorders (2%)
>1 (n=52)	M07 Psoriatic and enteropathic arthropathies (29%)	M08 Juvenile arthritis (3%)	E66 Overweight and Obesity (2%)	E11 Type 2 diabetes mellitus (2%)	D80 Immunodeficiency with predominantly antibody defects (2%)
Discontinued (n=125)	M07 Psoriatic and enteropathic arthropathies (21%)	C50 Malignant neoplasm of breast (5%)	K50 Crohn's disease (5%)	M05 Rheumatoid arthritis with rheumatoid factor (4%)	F32 Depressive episode (4%)
Conventional starters (n=3,297)					
0 (n=1,803)	M07 Psoriatic and enteropathic arthropathies (13%)	I10 Essential (primary) hypertension (4%)	E11 Type 2 diabetes mellitus (2%)	M05 Rheumatoid arthritis with rheumatoid factor (2%)	F32 Depressive episode (2%)
1 (n=474)	M07 Psoriatic and enteropathic arthropathies (4%)	I10 Essential (primary) hypertension (3%)	E11 Type 2 diabetes mellitus (2%)	L30 Other and unspecified dermatitis (2%)	E78 Disorders of lipoprotein metabolism and other lipidemias (1%)

**Subgroup**	**1st**	**2nd**	**3rd**	**4th**	**5th**
>1 (n=30)	M54 Low back pain, unspecified (3%)	M07 Psoriatic and enteropathic arthropathies (2%)	L30 Other and unspecified dermatitis (2%)	L26 Exfoliative dermatitis (1%)	M51 Other intervertebral disc disorders (1%)
Discontinued (n=1,620)	M07 Psoriatic and enteropathic arthropathies (8%)	I10 Essential (primary) hypertension (3%)	F33 Major depressive disorder, recurrent (2%)	L30 Other and unspecified dermatitis (2%)	K50 Crohn's disease (2%)
